# Intra- and inter-observer reliability of spinal inflection point localization: a cross-sectional radiographic study

**DOI:** 10.31744/einstein_journal/2026AO2130

**Published:** 2026-06-08

**Authors:** Guilherme Pianowski Pajanoti, Altacílio Aparecido Nunes, José Otávio Donadeli Tomé, Leonardo Gomes Baldoino, Lucas Dias Soares Silva, Luciano Miller dos Reis Rodrigues, Matheus Pippa Defino

**Affiliations:** 1 Hospital Israelita Albert Einstein São Paulo SP Brazil Hospital Israelita Albert Einstein, São Paulo, SP, Brazil.; 2 Department of Medicine Universidade Federal de São João del Rei Divinópolis MG Brazil Department of Medicine, Universidade Federal de São João del Rei, Divinópolis, MG, Brazil.; 3 Department of Orthopedics and Anesthesiology Faculdade de Medicina de Ribeirão Preto Universidade de São Paulo Ribeirão Preto SP Brazil Department of Orthopedics and Anesthesiology, Faculdade de Medicina de Ribeirão Preto, Universidade de São Paulo, Ribeirão Preto, SP, Brazil.

**Keywords:** Reproducibility of results, Observer variation, Spinal diseases, Diagnostic imaging, Radiography, Spinal

## Abstract

**Objective:**

To assess the intra- and inter-observer reliability of spinal inflection point localization on lateral radiographs of asymptomatic adults using a standardized measurement protocol, given its important role in sagittal alignment analysis and its potential influence on biomechanical interpretation and surgical planning.

**Methods:**

This cross-sectional study included 10 adults (five males and five females; age range 32–50 years) without spinal deformities or prior spinal surgery. Lateral and anteroposterior panoramic radiographs were obtained according to the institutional protocol. Three independent orthopedic residents with less than two years of experience assessed the cervicothoracic and thoracolumbar spinal inflection points using Surgimap^®^ software in three separate rounds, conducted at three-week intervals. The spinal inflection point was defined as the vertebral level at which curvature direction changes, according to the method described by Berthonnaud et al. Inter-observer agreement was evaluated using Cohen’s kappa index with 95% confidence intervals, and intra-observer reproducibility was assessed using the intraclass correlation coefficient (ICC).

**Results:**

The most frequent cervicothoracic spinal inflection point was C7–T1, whereas the most common thoracolumbar spinal inflection point was T12–L1. Inter-observer agreement for the cervicothoracic spinal inflection point ranged from kappa 0.19 (95% confidence interval: –0.15 to 0.55) to 0.41 (0.05–0.75), and intra-observer ICC ranged from 0.29 (0.07–0.54) to 0.56 (0.33–0.74). For the thoracolumbar spinal inflection point, inter-observer kappa ranged from –0.03 (–0.20 to 0.14) to 0.22 (0.01–0.44), whereas ICC ranged from 0.29 (0.08–0.53) to 0.56 (0.35–0.72). Wide confidence intervals suggested low precision, likely related to the small sample size and evaluator experience.

**Conclusion:**

In this sample, localization of the spinal inflection point by non-specialist evaluators showed low intra- and inter-observer agreement, indicating limited reliability and highlighting the need for standardized protocols or additional training.

## Highlights

■ Inflection point agreement was low to moderate among non-specialist raters.

■ Thoracolumbar junction showed the lowest inter-observer agreement.

■ Results support the need for protocol standardization and structured training.

## In Brief

Localization of the spinal inflection point on standing radiographs showed low to moderate intra- and inter-observer agreement among non-specialist evaluators. Variability was high at the cervicothoracic and thoracolumbar junctions, supporting the need for standardized protocols and structured training

## Graphical abstract

**Figure:**
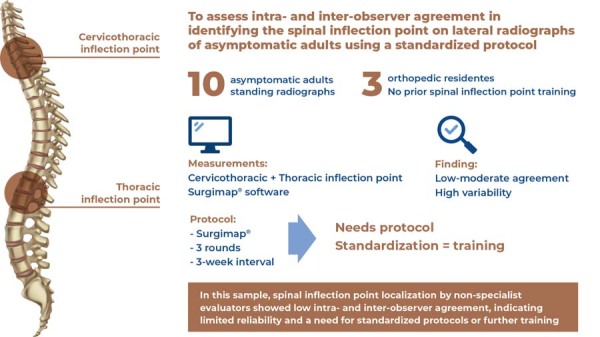

## INTRODUCTION

Evaluation of spinal curvature in the sagittal plane plays a central role in the diagnosis and management of spinal diseases.^(1)^ Numerous studies have demonstrated an association between sagittal alignment and favorable clinical outcomes.^(2)^ Traditionally, sagittal spinal curves are assessed using angular measurements obtained with the Cobb method and anatomical references based on the vertebral segment evaluated—cervical, thoracic, or lumbar. Thoracic kyphosis is measured as the angle between the superior endplate of T1 and the inferior endplate of T12, whereas lumbar lordosis is commonly measured as the angle between L1 and S1.^(1-4)^

However, these anatomical landmarks do not always reflect the true biomechanical transition of spinal curves. To address this limitation, Berthonnaud proposed a functional assessment of sagittal spinal curvature that prioritizes the inflection point—defined as the segment where the spinal curve changes direction—rather than fixed anatomical landmarks. Recent studies have shown that the inflection point rarely coincides with classical anatomical segments and may influence how spinal curves are classified and interpreted in clinical practice.^5^ This functional approach may have important implications for surgical planning, assessment of spinal balance, and prognosis, because improper segmentation may increase the risk of postoperative complications and unsatisfactory clinical outcomes.^(7)^

Despite its theoretical advantages, the use of the inflection point depends on adequate reproducibility and reliability among different observers. Previous studies have reported varying levels of agreement, often in populations that included more specialized raters and larger samples.^6^ However, limited data exist on determination of the spinal inflection point in routine clinical settings, particularly when evaluations involve non-specialist raters.

## OBJECTIVE

To assess the intra- and inter-observer reliability of spinal inflection point localization on lateral radiographs of asymptomatic adults using a standardized measurement protocol, given its important role in sagittal alignment analysis and its potential influence on biomechanical interpretation and surgical planning.

## METHODS

### Study design and ethics

This cross-sectional reliability study was conducted at *Hospital das Clínicas, Faculdade de Medicina de Ribeirão Preto, Universidade de São Paulo* (HC-FMRP-USP). The local Research Ethics Committee approved the study (CAAE: 87400125.5.0000.5440; #7.490.764). The requirement for informed consent was waived because the data were assessed retrospectively and anonymized.

### Sample selection

A convenience sample of 10 adults (five males and five females; age range 32–50 years) was included. The exclusion criteria included spinal diseases, congenital anomalies, vertebral deformities, prior spinal surgery, and poor-quality radiographic images. All participants were asymptomatic at the time of radiographic acquisition.

### Radiographic protocol

Full-length standing lateral panoramic spinal radiographs were obtained with participants in a standardized upright position with the arms supported, according to the institutional imaging protocol. Anteroposterior radiographs were also acquired to exclude coronal plane deformities and confirm the inclusion of patients with only sagittal plane changes. All images were acquired as part of routine clinical care.

### Inflection point determination

**Figure 1:**
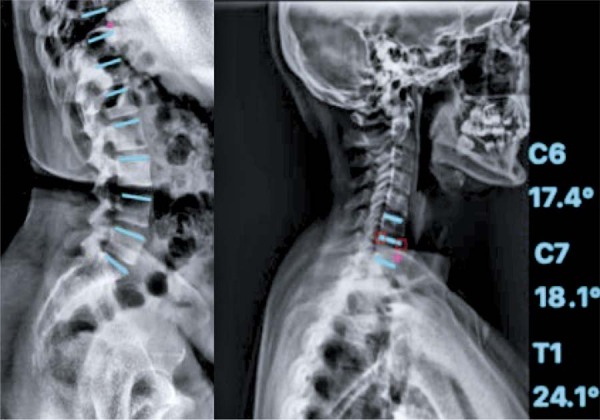
Radiographs demonstrating the inflection point at the thoracolumbar transition (left) and the cervicothoracic inflection point (right)

The spinal inflection point was defined as the vertebral level at which the sagittal curvature changes direction, specifically where adjacent angles indicate an increase in thoracic kyphosis or a decrease in lumbar lordosis. Measurements followed the method described by Berthonnaud et al. and were performed using Surgimap^®^ software (Nemaris Inc^™^), with use of the T1-slope tool when applicable. Examples appear in figure 1. For statistical analysis, vertebral levels were categorized according to intervertebral segments (C7–T1, T12–L1), and each segment was treated as a categorical variable.

### Observers

Three orthopedic residents in their first or second year of training independently performed the measurements. Each had less than two years of orthopedic experience and no prior formal experience in spinal radiographic analysis. Each observer performed the measurements in three separate sessions, with a three-week interval between sessions, and remained blinded to both their own previous results and those of the other observers. Prior to data collection, observers received a brief standardized instruction on the measurement protocol, without formal calibration or feedback.

### Statistical analysis

The intraclass correlation coefficient (ICC) with 95% confidence intervals (95%CIs) was calculated to assess intra-observer reproducibility. Inter-observer agreement was analyzed using Cohen’s kappa index with 95%CIs, considering both pairwise and overall agreement among the three raters. Both statistics are appropriate for categorical data reflecting agreement on vertebral levels. The level of significance was set at 5% (p<0.05). Statistical analyses were performed using SPSS.

## RESULTS

A total of 10 adults (five males and five females; age range 32–50 years) met the inclusion criteria and were evaluated (Table 1). All participants were asymptomatic and had no history of spinal disease, congenital anomaly, deformity, or prior surgery.

**Table 1:** Demographic and clinical characteristics of the study sample

Patient	Sex	Age (years)
1	M	32
2	F	34
3	M	36
4	F	37
5	M	39
6	F	42
7	M	44
8	F	45
9	M	48
10	F	50

Total n=10 (five males and five females). Age range 32–50 years.

Each of the three observers identified the cervicothoracic and thoracolumbar inflection points in three separate rounds, resulting in a total of 90 assessments (10 patients × 3 observers × 3 rounds).

The pooled distribution of inflection point determinations at both transitions, including all observers and rounds, appears in table 2. The segment C7–T1 represented the most frequently identified cervicothoracic inflection point, whereas T12–L1 represented the most common thoracolumbar inflection point.

**Table 2:** Distribution of inflection points by vertebral level across all raters and all rounds

Transition	Segment	Frequency (n)	Percentage (%)
Cervicothoracic	C6–C7	15	16.7
	C7–T1	28	31.1
	T1–T2	27	30.0
	T2–T3	7	7.8
	C5–C6	3	3.3
	C5–C7	2	2.2
	C4–C5	1	1.1
Thoracolumbar	T10–T11	3	3.3
	T11–T12	19	21.1
	T12–L1	36	40.0
	L1–L2	10	11.1
	L2–L3	4	4.4
	T9–T10	1	1.1
	T2–T11	1	1.1
	T10–T11	1	1.1
	T10–T11	1	1.1
	T10–T11	1	1.1

Pairwise inter-observer agreement for localization of the inflection point appears in table 3. For the cervicothoracic transition, kappa coefficients ranged from 0.19 (95%CI= –0.15 to 0.55) to 0.41 (95%CI= 0.05 to 0.75), indicating slight to moderate agreement. For the thoracolumbar transition, agreement was slight or absent, with kappa values ranging from –0.03 (95%CI= –0.20 to 0.14) to 0.22 (95%CI= 0.01 to 0.44). 95%CIs were wide for all comparisons, reflecting considerable variability and imprecision among observers.

**Table 3:** Cohen’s kappa for inter-observer agreement in localization of inflection points at cervicothoracic and thoracolumbar transitions

Pair of examiners	Transition	Kappa	95% confidence interval	Strength of agreement
1 *versus* 2	Cervicothoracic	0.41	0.05 to 0.75	Moderate
1 *versus* 3	Cervicothoracic	0.19	–0.15 to 0.55	Slight
2 *versus* 3	Cervicothoracic	0.22	–0.06 to 0.50	Fair
1 *versus* 2	Thoracolumbar	–0.03	–0.20 to 0.14	None
1 *versus* 3	Thoracolumbar	0.22	0.01 to 0.44	Fair
2 *versus* 3	Thoracolumbar	0.16	–0.03 to 0.34	Slight

Intra-observer reliability, calculated using the ICC, appears in table 4. For both transitions, ICC values for single measures were low (cervicothoracic: 0.30, 95%CI= 0.07–0.54; thoracolumbar: 0.29, 95%CI= 0.08–0.53). When the mean of the measurements was considered, ICC increased to 0.56 for both transitions, indicating moderate intra-observer consistency.

**Table 4:** Intraclass correlation coefficient (ICC) values for intra-observer reliability in localization of cervicothoracic and thoracolumbar inflection points, across all raters and all rounds

Transition	ICC (single measure)	95% CI	ICC (mean of measures)	95% CI
Cervicothoracic	0.30	0.07–0.54	0.56	0.19–0.77
Thoracolumbar	0.29	0.08–0.53	0.56	0.22–0.77

95%CI: 95% confidence interval.

Repeatability of inflection point localization for the cervicothoracic transition, stratified by patient and considering all raters and all rounds, appears in table 5. Percent agreement ranged from 66.6% to 100% across patients. Similar patterns were observed for the thoracolumbar transition and are summarized textually.

**Table 5:** Repeatability of cervicothoracic inflection point determination by patient, across all observers and all rounds

Patient	Agreement across rounds (%)	Most frequent segment
1	66.7	T1–T2/C7–T1
2	66.7	C7–T1
3	66.7	C7–T1/C6–C7
4	100	C7–T1
5	100	T1–T2
6	66.7	T1–T2/C7–T1
7	100	C6–C7/C7–T1
8	66.7	C7–T1
9	66.7	C6–C7/C7–T1
10	100	C6–C7

Overall, both inter- and intra-observer agreement for inflection point localization ranged from low to moderate, with most discordance occurring near anatomical transition segments. The findings indicate substantial variation both between observers and within individual observers, regardless of the anatomical region evaluated. Overall, the consistently low agreement across both spinal transitions highlights the limited reliability of inflection point localization using the current methodology.

## DISCUSSION

This study demonstrated low intra- and inter-observer agreement in the determination of inflection points at both the cervicothoracic and thoracolumbar transitions, supporting prior evidence that these functional landmarks often do not align with classical anatomical references.^6^ This pattern of divergence highlights the challenges associated with applying functional segmentation of the spine in the clinical assessment of sagittal balance and disease management.^(11,12)^

Our data identified C7–T1 as the most frequent inflection point at the cervicothoracic transition and T12–L1 at the thoracolumbar transition. Malka et al.^(10)^ similarly reported the highest frequency of inflection points at C7–T1, although the distribution ranged from T8 to L4, with 44% of cases located between C6–C7 and C7–T1. Park et al.^(11)^ observed a predilection for L2 and T1 levels, with age-associated distalization, whereas Vacari et al.^(6)^ identified T12–L1, particularly T12, as the most common thoracolumbar inflection point. Collectively, these studies and the present results indicate that inflection points rarely coincide with fixed anatomical landmarks.^(13,14)^

The shift from anatomical to functional spinal segmentation arises from the clinical importance of sagittal alignment parameters. As introduced by Berthonnaud et al.,^(4)^ functional segmentation defines lordosis as spinal segments in extension, kyphosis as segments in flexion, and the inflection point as the transition between these regions. Subsequent studies have reinforced this concept by demonstrating associations with overall sagittal alignment. This approach parallels determination of terminal vertebrae in the coronal plane, a method frequently used in planning deformity surgery.

Despite this conceptual advancement, the present findings, along with those of previous studies, consistently demonstrate limited reproducibility and precision in functional inflection point determination, particularly when performed by non-specialist raters with only basic radiographic training. The wide 95%CIs observed for kappa and ICC in this study further highlight substantial inter- and intra-observer variability, which represents a significant barrier to clinical adoption. Although the angular measurements themselves are not inherently complex, consistent identification of the inflection point may require not only clearly defined protocols but also improved training and possibly technological support.

Limitations of this study include the small sample size, the single-center convenience sample, and the limited experience of the observers. However, these characteristics reflect real-world settings in which such measurements are often performed by residents or physicians in training, thereby highlighting the practical challenges of incorporating functional segmentation into routine clinical workflows. Additional limitations include the absence of expert raters for comparison and the lack of correlation with clinical outcomes. Nonetheless, these factors represent relevant clinical scenarios and further emphasize the need for structured training and standardized protocols.

Future studies should expand these findings by including larger multicenter cohorts, incorporating experienced spine specialists as comparators, and exploring clinical correlations between inflection point determination and patient-reported outcomes. The application of automated image analysis and artificial intelligence, as suggested in recent literature, may further improve reproducibility and reduce observer dependence, thereby providing a more robust framework for integrating functional segmentation into both clinical care and research.

From a clinical perspective, variability in inflection point localization may directly influence sagittal alignment assessment and the interpretation of spinal biomechanics. Inconsistent identification of transition zones could impact surgical planning, particularly in defining fusion levels and alignment targets, potentially affecting postoperative outcomes. Therefore, improving the reliability of this parameter is essential before its incorporation into routine.

In summary, the results emphasize the discordance between anatomical and functional segmentation in inflection point determination and highlight the need for standardized, training-oriented, and potentially technology-assisted protocols before functional segmentation can be reliably incorporated into clinical or research workflows.

## CONCLUSION

Despite limitations related to sample size and observer experience, this study demonstrated poor agreement between anatomical and functional (dynamic) references in the determination of the spinal inflection point. In addition, intra- and inter-observer agreement for inflection point localization remained consistently low across methods and spinal transitions. These findings highlight the need for more standardized protocols and advanced measurement strategies before routine clinical or research use of functional spinal segmentation can be recommended. Reliable identification of the spinal inflection point is clinically relevant, as it may impact sagittal alignment assessment, biomechanical interpretation and surgical decision.
